# Subacute Presentation of Central Cord Syndrome Resulting from Vertebral Osteomyelitis and Discitis: A Case Report

**DOI:** 10.5811/cpcem.2019.8.44201

**Published:** 2020-04-23

**Authors:** Thomas Dang, Fanglong Dong, Greg Fenati, Massoud Rabiei, Melinda Cerda, Michael M. Neeki

**Affiliations:** * Arrowhead Regional Medical Center, Department of Emergency Medicine, Colton, California; † California University of Science and Medicine, Department of Emergency Medicine, San Bernardino, California

**Keywords:** central cord syndrome, vertebral osteomyelitis, discitis, case report

## Abstract

**Introduction:**

Central cord syndrome (CCS) is a clinical syndrome of motor weakness and sensory changes. While CCS is most often associated with traumatic events. There have been few documented cases being caused by abscesses resulting from osteomyelitis.

**Case Report:**

A 56-year-old male presented to a regional trauma center complaining of excruciating neck and bilateral upper extremity pain. Computed tomography of the cervical and thoracic regions revealed severe discitis and osteomyelitis of the fourth and fifth cervical (C4–C5) with near-complete destruction of the C4 vertebral body, as well as anterolisthesis of C4 on C5 causing compression of the central canal. Empiric intravenous (IV) antibiotic therapy with ampicillin/sulbactam and vancomycin was initiated, and drainage of the abscess was scheduled. After the patient refused surgery, he was planned to be transferred to a skilled nursing facility to receive a six-week course of IV vancomycin therapy. A month later, patient returned to emergency department with the same complaint due to non-compliance with antibiotic therapy.

**Discussion:**

Delayed diagnosis and treatment of osteomyelitis can result in devastating neurological sequelae, and literature supports immediate surgical debridement. Although past evidence has suggested surgical intervention in similar patients with presence of abscesses, this case may suggest that antibiotic treatment may be an alternative approach to the management of CCS due to an infectious etiology. However, the patient had been non-compliant with medication, so it is unknown whether there was definite resolution of the condition.

**Conclusion:**

In patients presenting with non-traumatic central cord syndrome, it is vital to identify risk factors for infection in a thoroughly obtained patient history, as well as to maintain a low threshold for diagnostic imaging.

## INTRODUCTION

Central cord syndrome (CCS) is a clinical syndrome of motor weakness and sensory changes that presents disproportionately greater in the upper extremities as compared to the lower extremities.^1^–^3^ While CCS is most often associated with traumatic events, there also have been a few documented cases being caused by other types of lesions such as demyelination or abscesses resulting from osteomyelitis.^4^ Cases of cervical osteomyelitis and the associated CCS are rare. We present a unique case of subacute CCS in a 56-year-old male with cervical vertebral osteomyelitis and discitis who recovered from his symptoms with conservative management and without any recommended surgical intervention.

## CASE REPORT

A 56-year-old male presented to a regional trauma center complaining of excruciating neck and bilateral upper extremity pain. He described a progressive weakness for the preceding month and specifically noted being unable to flex or abduct his bilateral shoulders without lying supine, necessitating assistance to complete activities of daily living. Patient history revealed poorly controlled type II diabetes mellitus, hepatitis C, methamphetamine use, and a history of remote intravenous (IV) heroin use. He reported progressively worsening generalized fatigue and constipation over the prior few months. In addition, he was experiencing mild persistent odynophagia without dysphagia. He denied any fevers, chills, bowel or bladder incontinence, shortness of breath, or focal weakness in the lower extremities.

On presentation, the patient was oriented and presented with a blood pressure 123/80 millimeters of mercury, heart rate 89 beats per minute, respiratory rate 18 breaths per minute, temperature 98.3° Fahrenheit, and oxygen saturation 99% on room air. Physical examination revealed tenderness to palpation of the cervical region midline at the levels of the C3–C5 spinous processes. No step off or any obvious abnormality was noted in the cervical region. Motor strength was noted to be 4+/5 in the bilateral upper extremities with decreased shoulder abduction and flexion, as well as decreased sensation to pinprick over the right C4 and C5 dermatomes. The rest of the physical and neurological exam was within normal limits.

Complete blood count revealed normal white blood cell count, elevated erythrocyte sedimentation rate of 52 millimeters per hour (mm/h) [normal range 0–15 mm/h], normal C-reactive protein level, and mild anemia with a hemoglobin of 12.5 grams per deciliter (g/dL) [normal range 13.5–17.5 g/dL] and hematocrit of 36.7 [normal range 41%–50%]. Additional tests provided evidence of poorly controlled diabetes, including 4+ glucose in urine, random serum glucose of 219 milligrams per deciliter, and HgbA1C of 9.7%.

**CPC-EM Capsule**

What do we already know about this clinical entity?

Central cord syndrome (CCS) is a clinical syndrome of motor weakness and sensory changes that can result in potentially devastating neurological sequelae.

What makes this presentation of disease reportable?

While CCS is associated with traumatic events, patients may present with subtle neurological deficits without an obvious inciting event.

What is the major learning point?

CCS requires timely diagnosis and treatment to prevent further neurological damage. In patients with risk for infection, early diagnosis may prevent increased morbidity.

How might this improve emergency medicine practice?

Early diagnosis of CCS caused by infection may allow alternative, non-surgical management that improves patient outcome.

**Image 1:**
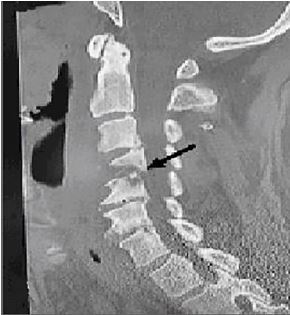
Computed tomography scan of sagittal plane of cervical spine showing fourth and fifth cervical osteomyelitis (arrow) upon admission.

**Image 2:**
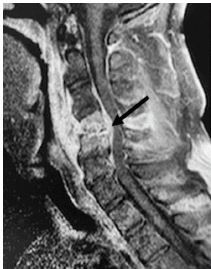
Magnetic resonance imaging of sagittal plane of cervical spine showing fourth and fifth cervical osteomyelitis (arrow) upon admission.

Computed tomography (CT) of the cervical and thoracic regions without contrast revealed evidence of severe discitis and osteomyelitis of C4–C5 with near-complete destruction of the C4 vertebral body, as well as anterolisthesis of C4 on C5 causing narrowing of the central canal (Image 1). Additionally, there appeared to be prevertebral, soft tissue swelling that was concerning for retropharyngeal abscess. Follow-up magnetic resonance imaging (MRI) with and without gadolinium of the cervical, thoracic, and lumbar spine confirmed the earlier CT findings of C4–C5 discitis and osteomyelitis with confirmation of surrounding prevertebral and epidural abscess (Image 2). In addition, MRI confirmed evidence of cord compression, although there was no signal change within the cord itself. Given these findings, the neurosurgery service recommended biopsy of the area around the cervical spine and to defer the initiation of antimicrobial therapy until after the biopsy was obtained.

Following the neurosurgery recommendation, interventional radiology was consulted to perform the biopsy but declined the procedure, citing risks associated with any interventions around the cervical spine. Subsequently, empiric IV antibiotic therapy of 3 gram (g) of ampicillin/sulbactam (every six hours) daily and 1.25 g of vancomycin twice daily was initiated for 10 days per infectious disease recommendations, and incision and drainage of the abscess with concurrent vertebral lesion biopsy was scheduled by neurosurgery.

On the day of the scheduled surgery, the patient stated that he no longer wished to proceed with the planned procedure. Given the patient’s refusal to proceed with surgical intervention, the decision was made to discharge him to a skilled nursing facility (SNF) with a peripherally inserted central catheter for a six-week treatment of IV antibiotics of vancomycin 1 g twice daily via peripherally inserted central line, along with strict cervical spine precautions that included cervical orthosis. Blood cultures remained negative throughout the course of the patient’s SNF stay, and infectious workup including chest radiograph, urine culture, and echocardiogram were noncontributory in identifying the primary source of infection. By the time of his discharge, 10 days later, the patient’s bilateral upper extremity weakness improved, and he reported decreased neck and arm pain.

**Image 3:**
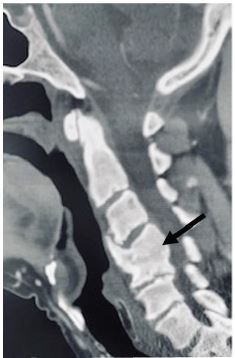
Computed tomography scan of sagittal plane of cervical spine showing fourth and fifth cervical fusion (arrow) two years after initial presentation.

About a month later, he once again returned to the emergency department with complaints of neck and back pain, which revealed he had been noncompliant with the antibiotic regiment previously prescribed. He refused any further workup and signed himself out against medical advice. Two years after the event, the patient presented to the same facility with complaint of chronic cervical pain, and a cervical CT without contrast revealed fusion of C4–C5 vertebrae (Image 3).

## DISCUSSION

Patients with CCS differ widely in presentation with varying sensory loss below the level of injury and may present with or without bladder dysfunction.^1^,^2^,^5^ It is most commonly associated with traumatic injuries, as approximately 9% of all traumatic spinal cord injuries result in CCS. It is particularly prevalent in older patients suffering cervical hyperextension injuries in the setting of high-velocity trauma.^1^,^2^,^5^–^8^ Despite its association with traumatic causes, there have been documented cases of CCS resulting from infection such as osteomyelitis and discitis.^4^

Osteomyelitis is a serious condition that rarely affects the spine, representing about 1% of all bone infections.^3^ Osteomyelitis presents a significant risk to patients, as infection can extend to a contiguous site, leading to discitis or abscesses.^9^ Among these incidents, cases confined to the cervical region represent only 3–6% of cases of spinal osteomyelitis.^3^ Despite their lower incidence, cervical infections are often associated with worse neurological outcomes, which may be related to the potential for more dynamic motion of the cervical spine compared to the thoracic or lumbar spine.^10^

There have been documented incidences of cervical epidural abscesses in literature, but very scant presentations with the subacute nature of this case of CCS (Table). Trombly and Guest documented a CCS secondary to an underlying vertebral osteomyelitis and epidural abscess, but the patient had the abrupt onset of symptoms secondary to minor trauma.^11^ Schimmer et al retrospectively studied 15 patients with osteomyelitis of the cervical spine and of the nine patients presenting with neurological symptoms, only one had been experiencing neurologic symptoms for at least two days – the remaining eight had sought medical attention within 24 hours of onset of neurological deficit.^3^ However, over a third of the patients had been experiencing worsening neck pain over several weeks with one of them experiencing pain for as long as four months, although these were present without coinciding neurological symptoms.^3^ Similarly, Khalid et al reviewed cases of upper cervical epidural abscesses, and few were associated with neurological symptoms that were subacute in onset.^12^ Of the cases that had at least two weeks of neurological symptoms prior to presentation, only one presented with CCS similar to the patient described above.^12^

Although rare, cases of pyogenic spinal vertebrae infections are on the rise, which is associated to increased life expectancy and higher prevalence of comorbidities with aging that result in immunosuppression.^13^,^14^ Misdiagnosis and delayed diagnosis can lead to devastating neurological sequelae, which is more significant when the cervical spine is involved; therefore, appropriate treatment should be initiated as soon as possible.^15^ Literature supports immediate surgical debridement in traumatic cases of osteomyelitis and epidural abscesses, even in the absence of neurological symptoms.^3^,^15^ While past studies suggest surgical intervention in this patient due to the presence of abscesses, he ultimately refused the procedure, and treatment consisting of antibiotic therapy provided initial relief of symptoms. However, it should be taken into consideration that the patient had been non-compliant with medication and follow-up, so it is unknown whether there was definite resolution of the condition. Nevertheless, this case may suggest that conservative antibiotic treatment may be an alternative approach to immediate surgical intervention in CCS due to infectious etiology.

## CONCLUSION

Pyogenic spinal vertebrae infections are rare, but they can present as a subacute cause of neurological symptoms, especially when the cervical spinal cord is affected. In patients presenting with non-traumatic central cord syndrome, it is vital to identify risk factors for infection in a thoroughly obtained patient history, as well as to maintain a low threshold for diagnostic imaging.

## Figures and Tables

**Table:** Summary of previous published case reports involving cervical spine abscess with sub-acute neurological symptoms, detailing age and gender, relevant comorbidities, level of infection, presentation, outcome, and duration of original onset.*

Author(s)	Age/gender	Relevant comorbidities	Level of infection	Presentation	Treatment	Outcome	Primary source of infection	Onset
Trombly and Guest, 2007^11^	60M	80 Pack-years smoking	C5–C7	Loss of sensation in arms bilaterally, unable to move arms or legs	Surgery, 6 Weeks of antibiotics	Independent walking in 2–3 weeks	None identified	One month of non-specific neurological symptoms
Schimmer et al, 2002^3^	65M	Unknown	C4–C5	Tetraplegia	Surgery	Continued complete neurological injury	Unknown	Tetraplegia for at least two days
Ahlback et al, 1970^12^	44F	Diabetes mellitus	C1–C2	Cervical pain, stiffness, limited ROM, neurological symptoms	Cervical collar, antibiotics	Residual cervical stiffness and limited ROM at 7-year follow-up	Left otitis media	6-weeks post-tonsillectomy
Zigler et al, 1987^12^	56F	Chronic renal failure, CHF	C1–C2	Hyperreflexia, positive Babinski sign	Soft collar, surgery	Full recovery, died shortly later due to CHF/Pneumonia	Cat scratch in left leg leading to septicemia	2 Weeks
Limbird et al, 1988^12^	61M	Hypertension, Renal Failure	C1–C2	Neck pain, central cord syndrome	Halo traction, antibiotics	Death secondary to myocardial infarction	None identified	3 Months
Azizi et al, 1995^12^	65M	Diabetes mellitus, cranial nerve abnormalities	Clivus-C1	Right ptosis, abducens nerve palsy, left facial weakness, cervical/shoulder pain	Halo neck stabilizer, antibiotics	Residual abducens palsy with otherwise full recovery	Left otitis externa	6 Months
Fukutake et al, 1998^12^	74M	Cervical spondylosis	C1–C2	Fever, cervical pain, difficulty ambulating, numbness in upper extremity	Antibiotics, surgery	Full resolution at 3 months	Post-TURP procedure, pneumonia	1 Month
Kuriomoto et al, 1998^12^	72F	Diabetes mellitus	C2	Afebrile, cervical pain and stiffness, right hemiparesis	Steroids, insulin, Antibiotics, Surgery	Right hemiparesis persisted	Non identified	2 Weeks
Yuceer et al, 2000^12^	72M	HIV	C2–C3	Neck pain and 4 limb weakness	Decompression and antibiotics	Full resolution by 6 months	Bilateral pneumonia	20 Days

* *M*, male; *F*, female; *C*, cervical; *ROM*, range of motion; *TURP*, transurethral resection of the prostate; *CHF*; congestive heart failure; *HIV*, human immunodeficiency virus.
