# This Article Corrects: “Subacute Presentation of Central Cord Syndrome Resulting from Vertebral Osteomyelitis and Discitis: A Case Report”

**DOI:** 10.5811/cpcem.2021.4.52958

**Published:** 2021-05-06

**Authors:** Thomas Dang, Fanglong Dong, Greg Fenati, Massoud Rabiei, Melinda Cerda, Michael M. Neeki, Sara Wattenbarger

**Affiliations:** * Arrowhead Regional Medical Center, Department of Emergency Medicine, Colton, California; † California University of Science and Medicine, Department of Emergency Medicine, San Bernardino, California; ‡ Kaweah Delta Medical Center, Department of Emergency Medicine, Visalia, California

*Clin Pract Cases Emerg Med.* 2020 May;4(2):267–271.

Subacute Presentation of Central Cord Syndrome Resulting from Vertebral Osteomyelitis and Discitis: A Case Report

Dang T, Dong F, Fenati G, Rabiel M, Cerda M, Neeki MM, Wattenbarger S

Erratum in

*Clin Pract Cases Emerg Med.* 2021 May;5(2):275. Author name and affiliation missing. The seventh author, Sara Wattenbarger, DO and her corresponding affiliations have been added.

Abstract

**Introduction:** Central cord syndrome (CCS) is a clinical syndrome of motor weakness and sensory changes. While CCS is most often associated with traumatic events. There have been few documented cases being caused by abscesses resulting from osteomyelitis.

**Case Report:** A 56-year-old male presented to a regional trauma center complaining of excruciating neck and bilateral upper extremity pain. Computed tomography of the cervical and thoracic regions revealed severe discitis and osteomyelitis of the fourth and fifth cervical (C4–C5) with near-complete destruction of the C4 vertebral body, as well as anterolisthesis of C4 on C5 causing compression of the central canal. Empiric intravenous (IV) antibiotic therapy with ampicillin/sulbactam and vancomycin was initiated, and drainage of the abscess was scheduled. After the patient refused surgery, he was planned to be transferred to a skilled nursing facility to receive a six-week course of IV vancomycin therapy. A month later, patient returned to emergency department with the same complaint due to non-compliance with antibiotic therapy.

**Discussion:** Delayed diagnosis and treatment of osteomyelitis can result in devastating neurological sequelae, and literature supports immediate surgical debridement. Although past evidence has suggested surgical intervention in similar patients with presence of abscesses, this case may suggest that antibiotic treatment may be an alternative approach to the management of CCS due to an infectious etiology. However, the patient had been non-compliant with medication, so it is unknown whether there was definite resolution of the condition.

**Conclusion:** In patients presenting with non-traumatic central cord syndrome, it is vital to identify risk factors for infection in a thoroughly obtained patient history, as well as to maintain a low threshold for diagnostic imaging.

PMCID: PMC7220002 [PubMed]

